# Integrative Profiling of Tumor and Blood Microenvironments to Uncover Molecular and Immune Determinants of Prognosis and Treatment Efficacy in Metastatic Colorectal Cancer

**DOI:** 10.3390/cancers18162651

**Published:** 2026-08-17

**Authors:** Elena Benidovskaya, Nicolas Huyghe, Maria Virginia Giolito, Pierre Coulie, Marc Van den Eynde

**Affiliations:** 1 Institut de Recherche Expérimentale et Cliniques, MIRO, UCLouvain, 1200 Brussels, Belgium; marc.vandeneynde@uclouvain.be; 2 De Duve Institute, Genes & Health, UCLouvain, 1200 Brussels, Belgium; nicolas.huyghe@uclouvain.be; 3 Institut de Recherche Expérimentale et Cliniques, FATH, UCLouvain, 1200 Brussels, Belgium; maria.virginia.giolito@uclouvain.be; 4 De Duve Institute, Immunity & Cancer, UCLouvain, 1200 Brussels, Belgium; pierre.coulie@uclouvain.be; 5 Digestive Oncology Department, Institut Roi Albert II, Cliniques Universitaires Saint-Luc, 1200 Brussels, Belgium

**Keywords:** metastatic colorectal cancer, tissue biomarkers, liquid biomarkers, immune microenvironment, genomic, transcriptomic

## Abstract

Metastatic colorectal cancer remains associated with poor prognosis despite major therapeutic advances, highlighting the need for robust biomarkers to refine treatment selection and monitor disease dynamics. This review summarizes emerging predictive and prognostic biomarkers in metastatic colorectal cancer across molecular and cellular layers, encompassing both tissue and circulating biomarkers. At the tissue level, we discuss genomic alterations and mutational signatures, transcriptomic classification systems and immune-related gene expression tools, protein-level immune checkpoint markers, and cellular determinants including immune infiltrates, cancer-associated fibroblasts and microbiome features. At the circulating level, we review biomarkers derived from liquid biopsy and peripheral blood, including circulating tumor DNA kinetics, T-cell receptor repertoire diversity, soluble cytokines and proteins, immune cell phenotyping, and circulating tumor cells. We highlight major challenges limiting clinical translation, including tumor heterogeneity, methodological variability, and the absence of standardized analytical pipelines and thresholds. Finally, we discuss future perspectives, emphasizing the integration of multi-omics biomarkers and artificial intelligence-driven strategies to improve biomarker validation and enable more precise management of metastatic colorectal cancer, particularly for patients with microsatellite-stable tumors who derive limited benefit from immune checkpoint inhibition.

## Simple Summary

Metastatic colorectal cancer is associated with poor prognosis, as patients respond very differently to available therapies, and the disease can change over time. Better biomarkers are needed to help clinicians choose the most effective treatment for each patient and to monitor whether therapy is working. This review summarizes the most promising biomarkers currently being investigated in metastatic colorectal cancer, including those measured in tumor tissue and those detected through blood-based tests. We describe prognostic and predictive markers related to tumor genetics, immune activity, tumor-associated cells, and interactions with the microbiome, as well as emerging metabolic and lipid-related signatures. We also highlight circulating biomarkers such as circulating tumor DNA, immune cell profiling, and circulating tumor cells. Finally, we discuss key limitations preventing clinical implementation and explore how combining multiple biomarker types with artificial intelligence may accelerate the development of reliable tools for personalized cancer treatment.

## 1. Introduction

Colorectal cancer (CRC) remains a major global health burden, ranking as the third most common cancer worldwide and the second leading cause of cancer-related death, largely attributable to the high incidence of metastatic disease and the unfavorable prognosis associated with advanced-stage disease [1]. Despite substantial efforts toward screening and early detection, up to 25–30% of patients are diagnosed with metastatic disease at presentation, and nearly 50% will develop metastases during the course of their disease, resulting in a 5-year relative survival rate of approximately 15% [2].

CRC arises through the stepwise accumulation of genetic and epigenetic alterations that transform normal colonic epithelium into invasive carcinoma. Although the classical adenoma–carcinoma sequence driven by *APC*, *KRAS*, *TP53* and *SMAD4* alterations accounts for most cases, alternative pathways involving mismatch repair deficiency or serrated lesions also contribute to colorectal carcinogenesis. These distinct evolutionary trajectories generate marked molecular heterogeneity, which underlies differences in prognosis, metastatic potential, and therapeutic response [3].

Metastatic colorectal cancer (mCRC) spreads most frequently to the liver, but also to the lung, peritoneum or distant lymph nodes. Local ablative and intra-arterial therapies represent standard treatment options for selected patients with liver metastases; however, only approximately 20% are eligible for potentially curative metastasectomy, and up to 80% will eventually experience disease relapse [4,5,6,7]. This is due, for a large part, to liver-specific immune tolerance owing to its constant exposure to gut-derived antigens via the portal circulation and to a specialized immune microenvironment dominated by tolerogenic antigen-presenting cells and immunosuppressive mediators (e.g., IL-10, TGF-β and PD-L1), which collectively limit T-cell activation while maintaining immune homeostasis [8,9,10,11].

The marked molecular heterogeneity of CRC has progressively reshaped the therapeutic landscape, with treatment selection increasingly relying on genomic biomarkers that predict sensitivity or resistance to targeted therapies [12]. Rat sarcoma viral proto-oncogene (*RAS*) hotspot mutations (*KRAS*/*NRAS*, ~50% of mCRC) are pivotal biomarkers that predict resistance to anti-Epidermal Growth Factor Receptor (EGFR) monoclonal antibodies and drive first-line treatment stratification. Patients with *RAS* wild-type (WT) tumors are eligible for anti-EGFR antibodies (cetuximab or panitumumab) combined with chemotherapy, whereas *RAS*-mutant patients are directed toward anti-Vascular Endothelial Growth Factor (VEGF)-based regimens or intensified triplet chemotherapy (FOLFOXIRI + bevacizumab), typically followed by de-escalation to maintenance therapy [12,13]. Notably, the *KRAS* G12C mutation, present in approximately 3–4% of mCRC, has spurred the development of specific covalent inhibitors, with sotorasib and adagrasib demonstrating clinical activity in pretreated patients when combined with anti-EGFR treatment [14,15]. *BRAF* V600E mutations (~10% of mCRC) confer poor prognosis and support the use of targeted therapies such as encorafenib plus cetuximab, combined with chemotherapy [16,17,18,19]. Primary tumor sidedness is a major prognostic and predictive factor guiding treatment decisions. *RAS*-WT patients with left-sided tumors tend to have a better prognosis and derive greater benefit from anti-EGFR therapy, whereas right-sided tumors are associated with worse outcomes and reduced anti-EGFR efficacy [12,20].

A small subset of mCRC patients (3–5%) harbor *HER2* amplification or overexpression, for which HER2-targeted therapy combination or antibody-drug conjugate demonstrated activity [21,22,23,24,25,26]. Among patients with high microsatellite instability (MSI-H)/deficient mismatch repair (dMMR) tumors, representing approximately 4–5% of metastatic cases, immune checkpoint inhibitors (ICIs) targeting PD-1 and CTLA-4 have shown substantial and sustained benefit of survival. In addition, serum CEA is routinely used for monitoring treatment response and disease progression, with both baseline levels and early kinetics carrying prognostic and predictive value [27].

The risk of relapses and chemotherapy resistance is further compounded by micro-metastatic disease. Notably, micro-metastases have been shown to display a predominantly stem-like, quiescent and differentiated phenotype, characterized by an inflammatory microenvironment and T cell exhaustion, limited fibrosis, and activation of metabolic programs associated with oxidative phosphorylation (OXPHOS) and fatty acid metabolism, consistent with metastatic dormancy. Since most conventional chemotherapeutic agents primarily target proliferating cells, these features may contribute to therapeutic resistance and subsequent disease recurrence [28,29].

Despite the availability of several predictive and prognostic biomarkers, current tools provide binary guidance and remain insufficient to accurately predict treatment response or detect resistance mechanisms, particularly in unresectable and treatment-resistant mCRC. Notably, the boundary between predictive and prognostic value is often blurred, as many biomarkers carry both functions depending on the therapeutic context. Furthermore, as the majority of mCRC tumors are microsatellite stable (MSS)/proficient mismatch repair system (pMMR), most patients derive limited benefit from ICIs, driving interest in identifying biomarkers of interest for potential immunotherapy-based combinations. Among these, immune-related biomarkers have emerged as one of the most extensively investigated classes owing to their central role in tumor progression and response to systemic therapies. Accordingly, this review focuses on tissue and circulating immune biomarkers, discussing them from the genomic to the cellular level.

This narrative review is based on a literature search performed primarily in PubMed using combinations of keywords related to metastatic colorectal cancer and tissue and circulating prognostic and predictive biomarkers. Original articles, clinical studies, and relevant reviews published in English over the past two decades were considered. Additional studies were identified through manual screening of reference lists, and biomarkers were selected based on biological rationale and the strength of available clinical evidence.

## 2. Tissue Biomarkers

**Figure 1:**
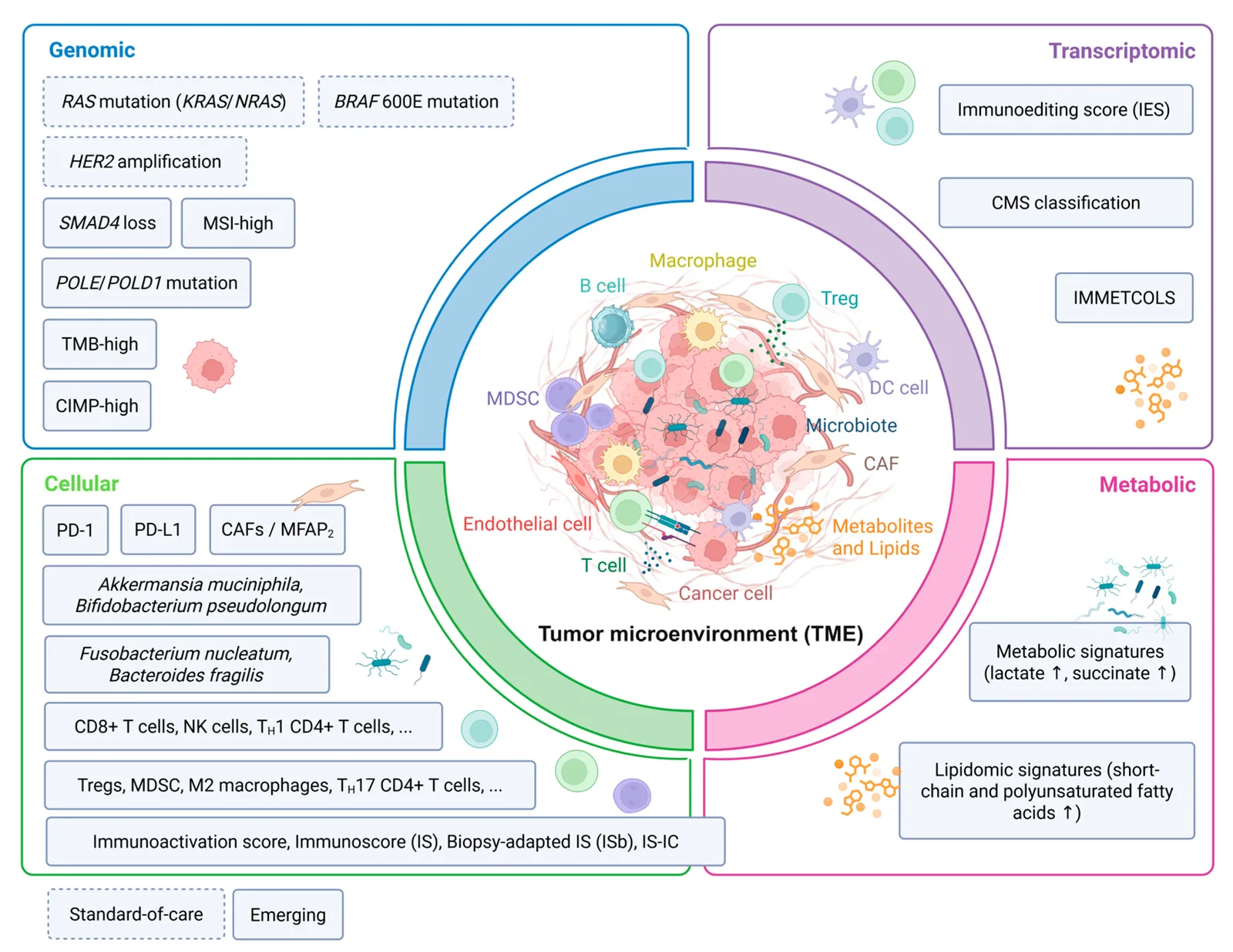
**Overview of the tissue mCRC biomarker landscape.** Biomarkers are organized by biological level. Clinical status is indicated for each biomarker category using dash outlines. BRAF—B-Raf proto-oncogene, serine/threonine kinase (e.g., BRAF V600E mutation). CAF—Cancer-associated fibroblast, CIMP—CpG island methylator phenotype, CMS—Consensus Molecular Subtype, DC—Dendritic cell, HER2—Human epidermal growth factor receptor 2 (ERBB2), IES—Immunoediting Score, IMMETCOLS—Immune-Metabolic Colorectal Cancer Subtypes, IS—Immunoscore, MDSC—Myeloid-derived suppressor cell, MFAP2—Microfibril-Associated Protein 2, MSI—Microsatellite Instability, NK cell—Natural Killer cell,, PD-1—Programmed Cell Death Protein 1, PD-L1—Programmed Death-Ligand 1, POLE—DNA Polymerase Epsilon Catalytic Subunit, POLD1—DNA Polymerase Delta 1 Catalytic Subunit, RAS—Rat sarcoma family of oncogenes, SMAD4—SMAD Family Member 4, T_H_1 cell—T helper type 1 cell, T_H_17 cell—T helper type 17 cell, TMB—Tumor Mutational Burden, TME—Tumor Microenvironment, Treg—Regulatory T cell.

Figure 1 and Table 1 provide an overview of the key concepts discussed in this section.

### 2.1. Genomic Level

#### 2.1.1. *SMAD4*

*SMAD4*, a tumor suppressor in the TGF-β pathway, is inactivated in 10–30% of CRC tumors, promoting tumor progression, metastasis, and chemotherapy resistance, making it a relevant prognostic biomarker [30,31,32]. In the REGOTORI trial, refractory mCRC patients with *SMAD4* alterations showed inferior outcomes when treated with regorafenib (multikinase inhibitor) plus toripalimab (anti-PD-1), with shorter PFS (1.6 vs. 2.4 months) and OS (5.1 months vs. not reached) [33,34].

#### 2.1.2. *POLE*/*POLD1*

*POLE* and *POLD1* mutations occur in ~1–2% of CRCs and impair DNA polymerase proofreading, leading to an ultra/hypermutated phenotype with very high tumor mutational burden and increased tumor-infiltrating lymphocytes, independent of MSI-H/dMMR [35,36,37]. These tumors show strong sensitivity to ICI, with significantly higher response rates and prolonged survival compared to non-mutated CRC, particularly when mutations affect the exonuclease domains [38]. Clinical studies reported meaningful activity of nivolumab and durvalumab (anti-PD1/-L1) in pretreated *POLE*/*POLD1*-mutated CRC (including MSS/pMMR), and retrospective data suggest even higher objective response rate (ORR) than for MSI-H/dMMR tumors, supporting *POLE*/*POLD1* mutations as predictive biomarkers for immunotherapy benefit [39,40,41].

#### 2.1.3. Tumor Mutational Burden

Tumor mutational burden (TMB) reflects the number of somatic mutations and leads to higher neoantigen load and better response to ICIs. In CRC, TMB-high tumors strongly overlap with MSI-H/dMMR (~97% of MSI-H/dMMR are TMB-H) [42,43,44,45]. While the FDA granted tumor-agnostic approval of pembrolizumab for TMB-High (≥10 mut/Mb) solid tumors based on KEYNOTE-158 [46], this approval was based on a dataset that included no CRC patients, limiting its applicability to mCRC. In MSS/pMRR mCRC, checkpoint inhibitors have failed to demonstrate meaningful clinical benefit at this threshold: patients with MSS/pMMR-TMB-H tumors treated with pembrolizumab achieved a median OS of only 4.5 months, compared to 33.6 months in MSI-H/dMMR-TMB-H patients [47], and the rare objective responses were restricted to tumors harboring *POLE*/*POLD1* mutations [38,48]. Together, these observations indicate that TMB alone is an insufficient biomarker for predicting response to ICIs or survival in mCRC, as its clinical relevance is highly dependent on the underlying molecular context.

TMB assessment also varies according to the analytical method used (WES, targeted NGS panels, or blood-based TMB), further complicating its clinical implementation. Although studies such as MyPathway [26] suggest that higher TMB may enrich for responders to atezolizumab, including a small subset of MSS/pMMR CRC, these observations support the use of TMB as part of a broader biomarker framework rather than as a standalone predictor of immunotherapy benefit [49,50,51,52]. These observations highlight, for the mCRC patients, the need to integrate the TMB into a broader panel of genomic and immune biomarkers to improve the stratification for immunotherapy.

#### 2.1.4. The CpG Island Methylator Phenotype

Epigenetic alterations in CRC include CpG island promoter hypermethylation, which silences tumor suppressor genes and defines the CpG island methylator phenotype (CIMP) which is associated with right-sided tumors, *BRAF* mutations, and often MSI-H features such as immune infiltration and PD-L1 expression [53,54,55]. To date, the CIMP status provides limited predictive value beyond MSI status and is generally linked to poor prognosis and reduced benefit from standard chemotherapy (oxaliplatin, irinotecan or fluorouracil) or anti-EGFR therapy in mCRC [56,57,58].

#### 2.1.5. Segment-Specific Molecular Heterogeneity Beyond Right/Left Classification

While the right/left dichotomy captures major biological differences, accumulating evidence indicates that CRC genomic alterations follow a continuum along the colon rather than a strict binary divide, with heterogeneity persisting even within each side. Next-generation sequencing of tumors from defined colonic segments has identified segment-specific mutational profiles that are not fully captured by sidedness alone, with *TP53*, *KRAS*, *BRAF*, *PIK3CA* and *CTNNB1* mutation frequencies varying progressively from cecum to rectum [120,121]. Within right-sided tumors, *RAS* mutation frequency declines and *BRAF* V600E frequency rises moving from cecal to hepatic flexure tumors, while within left-sided tumors, the sigmoid-rectal region shows fewer *PIK3CA*, *BRAF* and *CTNNB1* mutations and lower MSI than other left-sided sites, and transverse colon tumors cluster biologically closer to left- than right-sided disease [121]. This continuum extends beyond the genomic level: as discussed in Section 2.2, CMS distribution also shifts progressively along colon segments, with declining CMS1/CMS3 and rising CMS2 prevalence moving distally [121]. These findings support segment-level, rather than purely right/left, stratification as a more contemporary framework for interpreting predictive and prognostic biomarkers in CRC.

### 2.2. Transcriptomic Level

#### 2.2.1. Immunoediting Score

The Immunoediting Score (IES) integrates Genetic Immunoediting (GIE) determined by WES or whole genome sequencing (WGS) and the Immunologic Constant of Rejection (ICR) determined by RNA sequencing to quantify immune pressure on tumors and their capacity for immune evasion [59]. GIE measures neoantigen depletion, with lower ratios indicating strong immune selection and elimination of highly immunogenic clones, while higher GIE reflects weaker immune pressure [59,60]. ICR captures active anti-tumor immunity, including TH1-type inflammation, interferon signaling, cytotoxic effectors, and chemokines that recruit T cells [61,62]. Combining GIE and ICR, IES provides an integrated assessment of tumor–immune interactions: high IES tumors, with robust immune activity and retained neoantigens, are more likely to be controlled by the immune system, whereas low IES tumors exhibit immune evasion and progression. IES has shown prognostic value in both localized colon cancer [59], where it independently predicts disease-free survival and recurrence risk, and in metastatic colorectal cancer, where it correlates with overall survival outcomes [63]. Prospective validation of IES as a predictive biomarker for ICI response remains warranted.

#### 2.2.2. CMS Classification

The Consensus Molecular Subtypes (CMS) classify CRC into four transcriptomic groups [64]: CMS1 (“immune”, ~14%), enriched in MSI-H/dMMR, high TMB, strong immune infiltration and PD-1, CTLA-4 overexpression; CMS2 (“canonical”, ~37%), characterized by Wnt/MYC activation and an immune-desert microenvironment; CMS3 (“metabolic”, ~13%), with *RAS* mutations and limited immune activity; and CMS4 (“mesenchymal”, ~23%), displaying EMT, stromal infiltration, immunosuppressive populations (Tregs, M2 macrophages, MDSCs), and VEGF/TGF-β activation, conferring the worst prognosis [64,65,66]. Subtype-specific therapeutic associations have been proposed, and benefit reported for pembrolizumab or bevacizumab in CMS1, anti-EGFR in CMS2, and regorafenib plus nivolumab in CMS4 [67,68,69,70]. However, CMS application in metastatic disease remains debated, as the classification was derived from primary tumors and may be affected by spatial heterogeneity, tumor evolution, and inconsistent subtype assignment across lesions [63,71,72,73].

#### 2.2.3. IMMETCOLS

Since a substantial proportion of mCRC samples cannot be categorized into the current CMS, the IMMETCOLS signature was developed from transcriptomic profiling of CRC metastases and validated against TCGA datasets, stratifying tumors into three metabolic subtypes (IMC1-3) [74]. It should be noted that, as these classifications are transcriptomics-based, inferred metabolic programs may not fully reflect functional metabolic fluxes in vivo.

Most tumors (49–63%) fall into the epithelial IMC3 subtype, characterized by high glycolysis and OXPHOS with elevated Krebs cycle flux, clinically associated with extensive liver metastases and elevated LDH at diagnosis. The mesenchymal IMC1 subtype (20–36%) displays increased glycolytic activity, generating a hypoxic and acidic tumor microenvironment (TME), with ECM remodeling, strong CAF interactions, T-cell exhaustion, and IDO1/TDO2-mediated tryptophan degradation. The high lactate production in IMC1 tumors supports a potential benefit of MCT1 inhibitors (AZD3965 or diclofenac), which block lactate export and thereby limit TME acidification and its immunosuppressive consequences, combined with ICIs, with preclinical data showing reduced liver metastases and decreased CRC cell invasiveness [75]. Finally, IMC2 (13–19%) is characterized by glycolysis, glutaminolysis, enhanced fatty acid oxidation, and MHC-I degradation through autophagy, promoting immune evasion [74].

### 2.3. Cellular Level

The tumor microenvironment (TME) comprises a diverse array of immune and stromal cell populations that interact dynamically with tumor cells. Based on the degree of immune infiltration and activity, tumors are often broadly categorized as “immune-hot” or “immune-cold” [122]. These immune characteristics have therefore attracted considerable interest as potential biomarkers of treatment response, particularly in the context of immunotherapy.

#### 2.3.1. PD-1/PD-L1 Expressing Cells

PD-L1, expressed on tumor or immune cells, binds PD-1 on activated T, NK, and B cells, decreasing immune responses. Immunohistochemistry (IHC) for PD-L1 expression has been widely studied as a predictive biomarker for ICIs in solid tumors, including gastric, esophageal, and non-small cell lung cancer, but is dynamic and influenced by the TME, treatment, stage, and cytokines such as IFN-γ [76,77,78,79]. Assessment is further complicated by spatial heterogeneity, sampling, and assay variability [80].

In CRC, PD-L1 expression poorly correlates with MSI-H/dMMR status and does not reliably predict ICI response or survival [81,82]. Meta-analyses suggest PD-L1 on tumor cells shows heterogeneous prognostic significance, whereas PD-L1 on immune cells may correlate with better outcomes [83,84]. In MSS/pMMR mCRC, anti-PD-1/PD-L1 trials have consistently reported low ORRs independent of PD-L1 expression, with rates as low as 3% observed with anti-PD-L1 (atezolizumab) ± MEK inhibitor (cobimetinib) [85].

High PD-1-expressing cytotoxic T cells, particularly without CD4+ type-17 helper T (TH17) infiltration, may identify MSS/pMMR tumors with MSI-H/dMMR-like microenvironments responsive to ICIs [86,87,88]. Ongoing studies are exploring PD-1/PD-L1, T-cell composition, and gene expression as dynamic biomarkers. PD-L1 expression is assessed using the Combined Positive Score (CPS), calculated as the number of PD-L1-positive tumor cells, lymphocytes, and macrophages divided by the total number of viable tumor cells and multiplied by 100. Tumors with CPS ≥ 1 are considered PD-L1 positive [89]. In a trial combining anti-PD-1 (pembrolizumab) with anti-LAG-3 (favezelimab) in PD-L1+ mCRC, the ORR was 6.3% overall and 11.1% in PD-L1 CPS ≥ 1 tumors, with median OS 12.7 months and PFS 2.2 months [90].

#### 2.3.2. Immune Cells Infiltration in the Tumor

Multiple studies have explored immune-related biomarkers within the TME to better predict responses to immunotherapy and guide treatment strategies. The CRC TME is highly heterogeneous, encompassing diverse immune cell populations. Numerous studies have linked high infiltration by CD8+ cytotoxic T cells, TH1, follicular helper T cells, M1 macrophages, NK cells, and dendritic cells (DCs) with good prognosis in CRC. Conversely, high infiltration of MDSCs, B cells, M2 macrophages, and CD4+ TH17 cells is associated with poor prognosis. Notably, higher CD8+ T cell infiltration in tumors at baseline correlated with response to pembrolizumab plus azacitidine in patients with chemo-refractory proficient MMR (pMMR) mCRC [123,124,125,126].

##### Immunoscore

The Immunoscore, based on the density of CD3+ and CD8+ T cells in the tumor center and invasive margin, has emerged as a robust and reproducible measure of tumor immune infiltration. It provides strong prognostic value in localized CRC, where a high Immunoscore, defined as high infiltration of CD3+ and CD8+ T cells in the tumor center and invasive margin, is associated with significantly reduced risk of recurrence and improved survival, outperforming conventional clinicopathological parameters and supporting its integration into the Tumor Node Metastasis (TNM)-Immune classification systems [91,92].

In mCRC, even though a substantial spatial immune heterogeneity exists across lesions, a high Immunoscore, probably reflecting a pre-existing antitumor immune response, may predict benefit from ICIs, complementing established biomarkers such as MSI status [63,93,94]. Prospective validation remains limited but early clinical data are promising. The phase II POCHI trial (FFCD 1703) evaluated pembrolizumab combined with CAPOX and bevacizumab as first-line treatment in MSS/pMMR mCRC patients selected based on high immune infiltration (Immunoscore and/or TuLIS, evaluating the density and distance of CD3+ cells to the tumor invasive front [95]). Preliminary results demonstrated an objective response rate of 75%, a disease control rate of 96%, and a 12-month PFS of 68%, suggesting that immune infiltrate-based patient selection may identify a subset of MSS/pMMR mCRC patients who could benefit from ICI (NCT04262687) [96].

##### Biopsy-Adapted Immunoscore

Applying the Immunoscore to biopsies is challenging due to limited spatial representation, typically confined to the tumor core. To address this, a biopsy-adapted Immunoscore (ISb), based on CD3+ and CD8+ T-cell densities, has been developed and evaluated in locally advanced rectal cancer [97]. A high ISb correlated with enhanced cytotoxic immune activity, TH1-oriented gene expression, and improved histological response to chemoradiotherapy. Clinically, ISb-high patients showed significantly lower risk of relapse and death [97]. These findings were validated in an international cohort, where high ISb, defined by averaging the percentile ranks of CD3+ and CD8+ T-cell densities, with a mean percentile >0.70, was associated with markedly improved 5-year recurrence-free survival (91.3% vs. 62.5% and 53.1% for intermediate and low ISb, defined as a mean percentile < 0.25, respectively) and remained an independent predictor of recurrence and survival [98].

In the metastatic setting, the prognostic and predictive value of the biopsy-adapted ISb is supported by the prospective clinical trial AVETUXIRI. ISb stratified MSS/pMMR mCRC patients, treated with a combination of avelumab, cetuximab and irinotecan, into distinct prognostic groups, with ISb-high patients demonstrating significantly improved PFS compared to ISb-low (HR = 0.24, *p* < 0.01) and increased tumor shrinkage. A similar trend was observed for overall survival (OS), with prolonged survival in ISb-high patients. Importantly, a distance-based ISb (ISb_20), incorporating the spatial proximity between T cells and tumor cells, further refined patient stratification and was strongly associated with improved PFS (HR = 0.24, *p* = 0.004) and OS (HR = 0.20, *p* = 0.003). Unsupervised immune profiling identified an “immunogenic” cluster enriched in ISb-high tumors, characterized by increased density and proximity of activated PD-1+ T cells, and associated with favorable outcomes [63]. These results support ISb as a promising biomarker for immunotherapy benefit in mCRC, pending further validation in larger prospective trials.

##### Immunoscore-IC

The Immunoscore Immune-Checkpoint (Immunoscore-IC) extends the conventional Immunoscore by integrating immune checkpoint-related parameters to better capture tumor–immune interactions. It combines five features, including CD8+ T-cell density, spatial organization (e.g., clustering and proximity to PD-L1+ cells), and PD-L1+ cell density, generating a composite score that stratifies tumors into Immunoscore-IC-high (low risk) and Immunoscore-IC-low (high risk) groups. This approach refines both prognostic and predictive assessment beyond T-cell quantification alone and has demonstrated superior predictive performance compared to PD-L1 expression in solid tumors [99].

In mCRC, the clinical relevance of Immunoscore-IC has been highlighted in the AtezoTRIBE trial. In this phase II study, the addition of atezolizumab to FOLFOXIRI plus bevacizumab was associated with improved overall survival in the intention-to-treat population, with exploratory analyses showing a greater benefit in patients with Immunoscore-IC-high tumors. Notably, in the MSS/pMMR subgroup, Immunoscore-IC-high and TMB-high tumors showed the greatest benefit from atezolizumab, suggesting an additional value of combining immune contexture and genomic biomarkers [100]. Given those encouraging results, a follow-up study, AtezoTRIBE 2 (NCT06733038), is ongoing. However, conflicting evidence emerged from the ANICCA-Class II trial, which evaluated nivolumab in locally advanced/metastatic MSS/pMMR CRC. In this study, the Immunoscore-IC classification was not associated with predictive value for time on treatment, progression-free survival (PFS), or depth of response [101].

Given the lack of consensus on how to optimally integrate CD3, CD8, and PD-1/-L1 markers into composite immune scores, several studies have independently evaluated their predictive value in mCRC. In the REGOTORI trial, higher densities of stromal CD3+, CD3+CD8+, CD3+CD8−, and PD-1+CD3+ T cells were consistently associated with improved clinical outcomes, including prolonged PFS and higher disease control rates [33]. Increased infiltration of CD3+ and CD8+ T cells in the tumor stroma correlated with both longer PFS and a trend toward improved OS. Similarly, higher levels of PD-1+ and PD-1+CD3+ T cells in the stromal compartment were associated with prolonged PFS and favorable disease control, supporting their role as markers of an antitumor immune response. In contrast, PD-L1 expression, whether assessed as density or proportion of positive cells, did not show a consistent association with clinical outcomes [33].

Together, these findings suggest that quantitative and spatial assessment of T-cell subsets, particularly within the stromal compartment, may provide more robust predictive information than PD-L1 alone in mCRC, although standardization of these approaches remains a key challenge.

##### Immunoactivation Score

In the PanaMa trial, the predictive value of immune contexture was further explored in *RAS* wild-type mCRC patients receiving 5-fluorouracil/folinic acid ± panitumumab maintenance. Beyond confirming the prognostic relevance of the Immunoscore, specific immune subsets were identified as independent predictors of outcome: low CD163 and high PD-1 expression in the tumor center were associated with prolonged PFS, while high LAG-3 expression correlated with improved OS [102].

Panitumumab maintenance was associated with greater benefit in tumors exhibiting low levels of immunosuppressive markers and a favorable immune infiltration profile. By integrating multiple immune parameters including lymphocyte markers (CD3, CD8, CD45RO, FOXP3, CD20, granzyme B, and perforin), immune checkpoint markers (PD-1, PD-L1, IDO1, and LAG3) and the monocyte marker CD163, an “Immunoactivation score” was developed in patients for whom at least two predictive markers were available. This score identified a subgroup of patients who derived significant benefit from panitumumab, with improvements in both PFS (HR = 0.50; *p* < 0.001) and OS (HR = 0.54; *p* = 0.009) [102].

#### 2.3.3. Cancer-Associated Fibroblasts

Despite the major focus placed on tumor-infiltrating lymphocytes, the TME comprises a wide range of additional cellular components, many of which critically influence metastatic dissemination, treatment response, and disease progression. Among these, stromal cells, and particularly cancer-associated fibroblasts (CAFs), have emerged as key regulators of tumor biology. CAFs can be identified by the expression of fibroblast activation protein 1 (FAP1), largely absent in normal fibroblasts. Notably, the presence of FAP1-positive CAFs within the CRC tumor core has been associated with lymph node metastasis [103], highlighting this population as a promising target for anticancer therapeutic strategies. High CAF infiltration and enhanced crosstalk with tumor cells and T regulatory cells [74], via exosomes [104], have been associated with poor prognosis and reduced responsiveness to chemotherapy, chemoradiotherapy and immunotherapy [105,106,107]. Notably, CAFs can directly interact with CD8+ T cells and suppress their effector functions, notably through the expression of Microfibrillar-associated protein 2 (MFAP2) [108]. Therefore, MFAP2 has emerged as a potential biomarker of advanced disease stage and unfavorable clinical outcome [109,110].

#### 2.3.4. Cancer-Associated Microbiome

Emerging evidence indicates that tumors harbor distinct microbiomes that can influence cancer development, immune responses, and therapy outcomes. Nejman et al. analyzed 1526 non-gastrointestinal (GI) tumors using 16S rDNA sequencing and found tumor-type-specific microbiomes, primarily intracellular in cancer and immune cells, which correlated with tumor subtype and immunotherapy response [111].

In CRC, pathogenic bacteria such as *Fusobacterium nucleatum* (Fn) and *Bacteroides fragilis* (Bf) associate with MSI-H/dMMR status, *BRAF* mutations, right-sided tumors, poor chemotherapy response, and worse prognosis [112]. Fn is shared between primary tumors and metastases [113,114] and persists in metastases and xenografts, where metronidazole reduces bacterial load and tumor growth [114]. Fn retention after chemoradiotherapy in rectal cancer correlates with higher relapse and lower CD8+ T-cell infiltration, suggesting impaired antitumor immunity [115,116]. Fn has also been linked to immunotherapy response, potentially via modulation of PD-L1 expression and enhanced cytotoxic T-cell infiltration [117]. Thus, targeting Fn may potentially reduce the enrichment of these bacteria in CRC tissues and mitigate the pro-tumorigenic interactions between Fn and tumor cells [118]. *Prevotella intermedia* has also been linked with mCRC, promoting the invasion, migration, and ectopic tumorigenesis of CRC cells [114].

Overall, the CRC microbiome may influence immune cytotoxicity, tumor progression, and metastasis, although its role in metastatic CRC remains poorly defined [119]. The microbiota differs markedly between molecular CRC subgroups, with distinct bacterial communities characterizing MSI-H/dMMR andCMS1 tumors (high immunogenicity, BRAF-mutated, right-sided) and MSS/pMMR tumors (low TMB, immunosuppressed, left-sided).

Despite these promising findings, the clinical implementation of microbiome-based biomarkers remains challenging. Microbiome analyses are highly susceptible to contamination, particularly in low-biomass samples, where limited microbial DNA compromises sensitivity and increases the impact of technical artifacts. In addition, the lack of standardization in sample collection, DNA extraction, sequencing, and bioinformatic pipelines, together with confounding factors such as diet, antibiotic exposure, and host characteristics, limits reproducibility and cross-cohort validation. Harmonized methodologies and prospective multicenter studies will be essential before microbiome profiling can be reliably integrated into the clinical management of mCRC [111].

## 3. Circulating Biomarkers

Although tumor biopsies remain the clinical gold standard for diagnosis and assessment of prognostic and predictive biomarkers, they are invasive, not always feasible, and inherently limited by the tumor spatial heterogeneity. Circulating biomarkers could provide a less invasive and more cost-effective alternative, potentially affording a more global representation of both intra- and inter-tumoral heterogeneity across multiple lesions, while also enabling easier real-time monitoring of disease evolution and treatment response.

**Figure 2:**
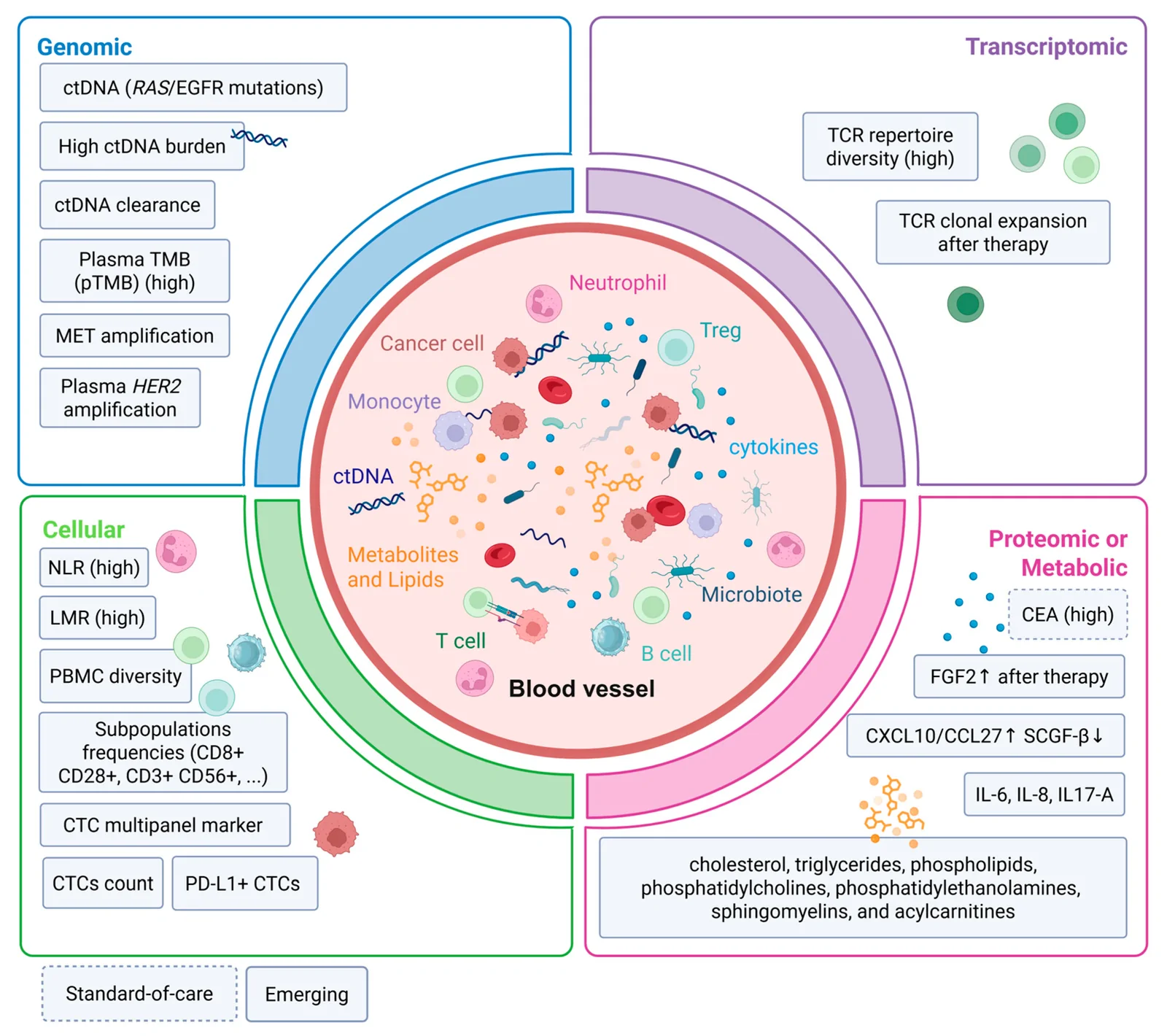
**Overview of the blood mCRC biomarker landscape.** Biomarkers are organized by biological level. Clinical status is indicated for each biomarker category using dash outlines. ctDNA—circulating tumor DNA, RAS—Rat Sarcoma (oncogene), EGFR—Epidermal Growth Factor Receptor, TMB—Tumor Mutational Burden, pTMB—Plasma Tumor Mutational Burden, MET—Mesenchymal–Epithelial Transition (proto-oncogene), HER2—Human Epidermal growth factor Receptor 2, TCR—T Cell Receptor, NLR—Neutrophil-to-Lymphocyte Ratio, LMR—Lymphocyte-to-Monocyte Ratio, PBMC—Peripheral Blood Mononuclear Cells, CD8+—Cluster of Differentiation 8 (cytotoxic T cells), CD28+—Cluster of Differentiation 28 (co-stimulatory molecule), CD3+—Cluster of Differentiation 3 (T cell marker), CD56+—Cluster of Differentiation 56 (NK cell marker), CTC—Circulating Tumor Cell, PD-L1—Programmed Death-Ligand 1, Treg—Regulatory T cell, CEA—Carcinoembryonic Antigen, FGF2—Fibroblast Growth Factor 2, CXCL10—C-X-C Motif Chemokine Ligand 10, CCL27—C-C Motif Chemokine Ligand 27, SCGF-β—Stem Cell Growth Factor Beta, IL-6—Interleukin 6, IL-8—Interleukin 8, IL-17A—Interleukin 17A.

Figure 2 and Table 2 provide an overview of the key concepts discussed in this section.

### 3.1. Genomic Level: Circulating Tumor DNA (ctDNA)

Liquid biopsies, particularly ctDNA, provide a minimally invasive tool to track genomic alterations, tumor burden, and acquired resistance mechanisms in CRC. ctDNA alterations in the EGFR pathway associate with resistance to anti-EGFR therapy [127,128], and longitudinal ctDNA *RAS* mutation burden correlates with radiological tumor burden measurements [129]. Notably, in the CO.26 trial, plasma TMB, but not tissue TMB, predicted benefit from durvalumab plus tremelimumab in mCRC, supporting the relevance of ctDNA-based immune biomarkers, and several ongoing trials are investigating ctDNA dynamics as early indicators of ICI response or resistance [130,131,132].

ctDNA kinetics have demonstrated prognostic value across multiple treatment settings: in the Valentino trial, including mCRC *RAS* WT patients treated with anti-EGFR, baseline ctDNA was associated with progression risk while ctDNA clearance correlated with deeper responses [133,134]; in SAMCO-PRODIGE 54, focusing on MSI-H/dMMR mCRC patients, ctDNA changes were strongly associated with PFS and OS, particularly in the avelumab arm [135]. Higher plasma *HER2* amplification was associated with improved outcomes in DESTINY-CRC01 [25,129] and HERACLES [21], and high baseline cell free DNA (cfDNA)/ctDNA consistently correlated with poorer OS in regorafenib- and chemotherapy-treated cohorts [136,137]. MET amplification in cfDNA has also shown predictive value in cabozantinib plus panitumumab-treated patients [138]. In early-stage disease, baseline cfDNA was independently associated with DFS in patients treated with preoperative FOLFOX chemotherapy [139,140], while the ongoing COPERNIC trial investigates the predictive value of early on-treatment ctDNA dynamics for treatment response in chemotherapy-refractory mCRC [141].

### 3.2. Genomic and Transcriptomic Level

The peripheral blood T cell receptor (TCR) repertoire is being investigated as a potential biomarker of immune response to ICIs. Higher TCR diversity has been associated with improved clinical outcomes in melanoma and is currently under investigation in CRC [142].

In mCRC, T cell diversity has been reported to be lower than in primary tumors [73] and in healthy controls [143]. However, other studies suggest that TCR diversity may be higher at metastatic sites than in primary tumors, potentially reflecting an increased infiltration of γδ T cells. γδ T cells are unconventional T lymphocytes expressing a γδ T-cell receptor instead of the classical αβ TCR, enabling rapid immune surveillance and antitumor responses [144]. The enrichment of γδ T cells in metastatic tumors, together with their correlation with tumor burden, supports their proposed role in metastatic immune surveillance [145].

Longitudinal analyses have shown that TCR repertoire diversity and complementarity-determining region 3 (CDR3) clonotype diversity decrease in most mCRC patients following therapy, consistent with the clonal expansion of tumor-specific T cells and associated with improved tumor response, including lower CEA levels and reduced tumor size [143]. Baseline TCR diversity has also been significantly associated with partial response to chemotherapy (OR = 5.29, *p* = 0.04) [143]. Moreover, TCR diversity may help predict response to ICIs targeting PD-L1 and CTLA-4 [146]. Conversely, low baseline diversity has been linked to postoperative relapse [73].

**Table 2:** Emerging circulating biomarkers in mCRC.

Biomarker	Biological Level	Type	Value	Key Finding	Validation Status	References
ctDNA: *RAS*/EGFR pathway mutations	Genomic	Cell-free circulating tumour DNA	Predictive (anti-EGFR resistance); Prognostic	RAS mutations in blood associated with anti-EGFR resistance; ctDNA RAS burden correlates with radiological tumour burden (SLD)	Prospective trials; longitudinal monitoring validated (Valentino, REMARRY, C-PROWESS)	[127,128,129]
ctDNA: MSI-H status and plasma TMB (pTMB)	Genomic	Cell-free circulating tumour DNA	Predictive (ICI)	Plasma TMB (clonal and subclonal) predictive of improved outcomes with durvalumab + tremelimumab, whereas tissue TMB was not	Single prospective trial; larger validation needed (CO.26)	[130,131,132]
ctDNA kinetics (clearance/early dynamics)	Genomic	Cell-free circulating tumour DNA	Prognostic; Predictive (treatment monitoring)	ctDNA clearance associated with deeper tumour response (Valentino); ctDNA changes strongly associated with PFS and OS in immunotherapy arm (SAMCO-PRODIGE 54)	Multiple prospective trials; ongoing validation (Valentino, SAMCO-PRODIGE 54, COPERNIC)	[133,134,135,139,140,141]
Plasma *HER2* amplification	Genomic	Cell-free circulating tumour DNA	Predictive (anti-HER2)	Higher plasma HER2 amplification associated with improved response and longer PFS with HER2-targeted therapy	Prospective; limited to HER2+ subgroup (DESTINY-CRC01, HERACLES)	[21,25,129]
MET amplification (ctDNA)	Genomic	Cell-free circulating tumour DNA	Predictive (investigational)	MET amplification associated with response to cabozantinib + panitumumab	Preliminary single case series/exploratory cohort; prospective validation absent	[138]
TCR repertoire diversity	Transcriptomic	T cell receptor sequencing (CDR3 clonotypes)	Prognostic; Predictive (ICI, investigational)	Decreased TCR diversity post-therapy, consistent with clonal expansion of tumour-specific T cells, associated with improved tumour response (OR = 5.29, *p* = 0.04); low baseline diversity linked to postoperative relapse	Retrospective longitudinal analyses/exploratory cohort; prospective validation lacking	[73,142,143,144,145,146]
IL-6	Proteic	Cytokine	Prognostic	Independently prognostic in unresectable mCRC; elevated in patients with liver or lung metastases; CISIG-positive status predicts shorter OS (13.5 vs. 25.0 months)	Retrospective; not validated as standalone clinical biomarker (CISIG)	[147,148,149]
IL-8	Proteic	Cytokine	Prognostic	Elevated IL-8 linked to poor prognosis (HR = 1.54) and stage IV disease (HR = 2.28); elevated pre-chemotherapy IL-8 correlates with disease progression	Retrospective/correlative; phase I targeting showed no ORR (Phase I (HuMax-IL8))	[147,149,150,151,152]
CXCL10/CTACK (CCL27)	Proteic	Chemokine	Prognostic	Lower CXCL10 or higher CCL27 levels associated with improved DFS and OS	Retrospective; limited evidence	[148]
Neutrophil-to-lymphocyte ratio (NLR)	Cellular	Inflammation ratio	Predictive (limited); Prognostic	Fair predictive ability for early treatment response with pembrolizumab + chemotherapy; not significantly associated with PFS	Retrospective; widely studied but inconsistent cutoffs	[153]
Lymphocyte-to-monocyte ratio (LMR)	Cellular	Inflammation ratio	Prognostic (trend)	Higher LMR associated with trend toward improved PFS; slightly lower predictive performance than NLR for early response	Retrospective; limited prospective data	[153]
PBMC diversity	Cellular	Immune cell diversity (flow cytometry)	Predictive (investigational)	PBMC diversity correlated with response after 3 cycles of chemotherapy + avelumab (R^2^ = 0.14, *p* = 0.036); not significant at baseline	Single prospective trial (AVETUX); exploratory	[154]
Circulating tumour cells (CTCs) count	Cellular	Epithelial circulating tumour cells	Prognostic	≥1 CTC/7.5 mL associated with shorter PFS and OS; independent prognostic factor for OS (HR = 3.14); high-volume CTCs (>3) associated with reduced OS (HR = 3.97)	Prospective; cutoff variability remains a limitation	[155,156,157]
PD-L1 expression on CTCs	Cellular	Immune checkpoint on circulating tumour cells	Predictive (regorafenib response)	PD-L1+ CTCs mainly in partial response/stable disease; PD-L1− CTCs associated with progressive disease; median PFS 6.3 vs. 2.1 months	Retrospective; single cohort; requires prospective validation	[158,159]
CTC marker panel (CD45, GAPDH, VIL1, TIMP1, CLU, SNAIL1/2, TWIST1, LOXL2/3, ZEB1/2, E47)	Cellular	Multimarker CTC signature	Prognostic; treatment monitoring	High baseline expression associated with shorter PFS (6.3 vs. 12.7 months) and OS (12.7 vs. 24.2 months); identified refractory patients missed by CT imaging	Retrospective; single cohort	[160]

Abbreviations: ctDNA, circulating tumor DNA; cfDNA, cell-free DNA; pTMB, plasma tumor mutational burden; MSI-H, microsatellite instability-high; ICI, immune checkpoint inhibitor; SLD, sum of longest diameters; HER2, human epidermal growth factor receptor 2; TCR, T cell receptor; CDR3, complementarity-determining region 3; OR, odds ratio; IL, interleukin; CXCL10, C-X-C motif chemokine ligand 10; CTACK, cutaneous T-cell-attracting chemokine; CCL27, C-C motif chemokine ligand 27; FGF-2, fibroblast growth factor 2; DFS, disease-free survival; PFS, progression-free survival; OS, overall survival; HR, hazard ratio; ORR, objective response rate; NLR, neutrophil-to-lymphocyte ratio; LMR, lymphocyte-to-monocyte ratio; PBMC, peripheral blood mononuclear cell; NKT, natural killer T cell; CTC, circulating tumor cell; PD-L1, programmed death-ligand 1; CT, computed tomography; CISIG, circulating inflammatory signature.

### 3.3. Protein Levels in Serum

Cytokines including interleukin (IL)-6, IL-8, and IL-17A modulate immune responses in CRC. These mediators have been implicated in systemic inflammation, primarily through neutrophil activation, and are known to promote angiogenesis, tumor progression, and metastasis and modulate immune responses in CRC. Elevated IL-8 is linked to poor prognosis (HR = 1.54, 95% CI 1.03–2.32) and stage IV disease (HR = 2.28, 95% CI 1.60–3.25) [150]. Serum IL-6 and IL-8 are higher in patients with liver or lung metastases [147], and elevated IL-8 before chemotherapy correlates with disease progression [151]. A phase I study targeting IL-8 with HuMax-IL8 (BMS-986253) showed no objective responses, though 73% of patients had stable disease [152]. IL-17A inhibition enhances PD-1 blockade efficacy in preclinical models [161].

Lower IL-6, stem cell growth factor beta (SCGF-β), and CXCL10 (T-cell chemoattractant, linked to TH1/IFNγ-induced inflammation) or higher cutaneous T-cell-attracting chemokine (CTACK or CCL27) levels are associated with improved DFS and OS [148]. Combining cytokines into a circulating inflammatory signature (CISIG) further improves prognostic value. In mCRC patients, CISIG-positive status (high circulating levels of ≥2 of the 3 inflammation markers among miR-21, IL-6, and IL-8) was associated with shorter relapse-free survival and OS in resected patients (RFS 18.4 vs. 31.4 months; OS 46.2 vs. 66.0 months) and OS in unresected mCRC patients (OS 13.5 vs. 25.0 months), with IL-6 as an independently prognostic marker in unresectable disease [149].

Other circulating proteins, such as fibroblast growth factor-2 (FGF-2), may also inform prognosis. In FOLFIRI/bevacizumab-treated mCRC patients, increases in FGF-2 during therapy were associated with longer median PFS (12.85 vs. 7.57 months; HR = 0.73, 95% CI 0.43–1.27) [162].

### 3.4. Circulating Cells

#### 3.4.1. Peripheral Blood Circulating Cells

Systemic inflammation, as reflected in peripheral blood parameters, plays a key role in cancer progression and can provide accessible biomarkers for disease monitoring, survival prediction, and response to treatment. Among the most widely investigated inflammation-related biomarkers are blood-derived ratios such as the neutrophil-to-lymphocyte ratio (NLR) and the lymphocyte-to-monocyte ratio (LMR), which integrate information on both pro-tumoral inflammatory components and anti-tumoral immune competence.

In mCRC patients treated with pembrolizumab plus chemotherapy, NLR demonstrated a fair ability to identify early treatment response, while LMR demonstrated a slightly lower predictive performance. However, NLR was not significantly associated with PFS, whereas higher LMR levels showed a trend toward improved PFS [153].

Beyond these global inflammation ratios, peripheral blood immune profiling suggests that immune diversity and immune cell composition may capture more subtle treatment-induced changes. Notably, the AVETUX trial reported a correlation between peripheral blood mononuclear cell (PBMC) diversity and response after three cycles of chemotherapy plus avelumab (R^2^ = 0.14, *p* = 0.036), whereas no significant association was observed at baseline (R^2^ = 0.09, *p* = 0.096), suggesting that therapy-driven immune remodeling may characterize responder patients [154].

Flow cytometry and mass cytometry further provide mechanistic insights into circulating immune cell dynamics. In CRC, increased TH1/CD4+ T cell frequencies have been associated with ICI response, while higher pre-treatment regulatory T cell levels have been linked to non-response [163]. Additionally, mCRC patients were reported to display an increased abundance of CD152+CD14+ monocytes, and mortality correlated with the presence of CD141-CD1c+ myeloid dendritic cells and CD4-CD8- conventional T cells [164]. Higher circulating CD8+CD28+ T cell counts were associated with improved chemotherapy responses in mCRC, including higher ORR [165]. Finally, the AVETUXIRI trial showed that while major immune cell populations remained stable in blood, changes in specific T- and NK-cell subsets may be associated with different survival outcomes. A decrease in TH17 frequency over time was associated with poorer outcomes, whereas higher baseline NKT frequency and an increase over time were associated with improved tumor response, PFS, and OS [166].

#### 3.4.2. Circulating Tumor Cells

Circulating tumor cells (CTCs) carry significant prognostic value in mCRC: detection of ≥1 CTC per 7.5 mL blood was independently associated with shorter PFS and OS (HR = 3.14, *p* = 0.021) [155], high CTC counts (>3) are associated with reduced OS (HR = 3.97) and DFS (HR = 2.55) [156], and CTC levels correlate with TNM staging and tumor burden [157].

Beyond enumeration, PD-L1 expression on CTCs adds predictive value: PD-L1+ CTCs are associated with improved PFS (6.3 vs. 2.1 months) and OS (9.1 vs. 2.5 months), and in regorafenib-treated patients, PD-L1+ CTCs were enriched in responders while PD-L1− CTCs correlated with progressive disease [158]. Conversely, nuclear PD-L1 expression on CTCs was linked to worse OS (HR = 2.437, *p* = 0.026) [159].

Finally, a multi-marker CTC signature measured by qualitative polymerase chain reaction (including epithelial mesenchymal transition markers GAPDH, VIL1, CLU, TIMP1, LOXL3, ZEB2) showed strong prognostic value in patients receiving fluoropyrimidine-based regimens, with high baseline expression or increasing levels on treatment associated with markedly shorter PFS and OS. Notably, this signature identified therapy-refractory patients missed by conventional imaging [160].

## 4. Limits, Challenges and Perspectives

Substantial research effort is currently devoted to improving patient stratification, with tissue-based biomarkers remaining the gold standard but subject to well-known limitations: invasiveness, sampling feasibility, and crucially, the inability of a single biopsy to capture intra- and inter-tumoral heterogeneity [167,168,169]. Circulating biomarkers offer a minimally invasive alternative, better reflecting this heterogeneity and are particularly valuable for dynamic monitoring of treatment response and relapse detection [170].

As highlighted, immune biomarkers represent the most extensively investigated category, with particularly promising results for the Immunoscore and ctDNA kinetics across multiple clinical settings [21,63,92,135,136,137,138]. However, MSS/pMMR status and the high prevalence of liver metastases in mCRC restrict ICI benefit [11]. Moreover, the intrinsically tolerogenic hepatic microenvironment limits the applicability of immune infiltration-based biomarkers [10], highlighting the need for orthogonal biomarker strategies.

In this context, metabolic and lipidomic biomarkers emerge as promising complementary avenues, both at the tissue and circulating levels. Colorectal cancer is characterized by profound metabolic reprogramming involving glycolysis, amino acid metabolism, redox homeostasis, and lipid remodeling, with recurrent alterations in metabolites such as lactate, succinate, glutamate, taurine, glutathione, uracil, hypoxanthine, and polyamines [148,171,172,173,174,175,176].

Several tissue-based studies have demonstrated the clinical relevance of these alterations, identifying metabolite signatures associated with tumor recurrence, overall survival, and metastatic dissemination, while lipidomic analyses further revealed stage-dependent changes in fatty acids, acyl-carnitines, glycerophospholipids, and cholesteryl esters that discriminate early from advanced disease [171,174,176,177,178]. These findings are supported by growing evidence linking dysregulated lipid metabolism to tumor progression through altered fatty acid uptake and β-oxidation, microbiota-associated changes in short-chain fatty acids, and imbalances between pro- and anti-inflammatory polyunsaturated fatty acids [179,180,181,182,183,184,185,186].

Importantly, several of these metabolic alterations are also detectable in circulation. In patients with metastatic CRC, circulating metabolite profiles reflecting ketone body production, amino acid depletion, membrane lipid remodeling, and enhanced fatty acid oxidation have been associated with treatment response and survival [148,187,188,189,190,191,192]. For example, elevated 3-hydroxybutyrate levels were linked to poorer disease-free survival and preferentially increased in non-responders to chemotherapy, whereas higher histidine concentrations were associated with improved outcomes [148,187,188,189]. Likewise, circulating lipid markers, including cholesterol, triglycerides, phospholipids, phosphatidylcholines, phosphatidylethanolamines, sphingomyelins, and acylcarnitines, have shown associations with disease progression and prognosis [148,190,191,192]. Collectively, these studies suggest that metabolic and lipidomic profiling may capture key biological processes underlying tumor progression and treatment response, highlighting their potential as complementary biomarkers for patient stratification and outcome prediction in metastatic CRC.

While these findings highlight the promising potential of metabolomic and lipidomic profiling for patient stratification and outcome prediction, several practical considerations must be acknowledged before their clinical translation. Unlike proteomics or transcriptomics, many metabolites of interest are chemically labile and prone to rapid degradation, making sample acquisition speed, handling conditions, and storage protocols critical determinants of data quality. The choice of biological matrix further adds complexity: metabolic and lipidomic profiles differ substantially between tumor tissue, adjacent normal tissue, plasma, serum, and stool, and findings derived from one compartment may not be directly transferable to another. Tumor-derived metabolic alterations may be diluted or confounded in circulation, while stool-based analyses capture both host and microbiota-derived signals, requiring careful interpretation. Beyond pre-analytical factors, metabolic and lipidomic signatures are highly sensitive to extrinsic biological variables, particularly dietary habits and concomitant medications. Nutritional status, dietary composition, and short-term food intake can profoundly reshape circulating and tissue metabolite profiles, while chemotherapy agents, targeted therapies, and supportive medications may independently alter lipid metabolism, amino acid turnover, and redox homeostasis, potentially confounding the interpretation of treatment-associated metabolic changes. Controlling these variables in study design and statistical analyses is therefore essential yet rarely performed systematically in current biomarker studies. Analytical platform variability, across mass spectrometry methods, chromatographic approaches, and data processing pipelines, further limits cross-study comparability. Collectively, the lack of standardized pre-analytical and analytical protocols, combined with the biological lability of key metabolites, the diversity of sampling strategies, and the substantial influence of diet and treatment on metabolic profiles, represents a major barrier to the reproducibility and clinical implementation of metabolomic and lipidomic biomarkers in mCRC.

Despite these advances, there is still no clear consensus, and studies often report divergent findings focused on different biological aspects, including immune response, metabolism, and the tumor microenvironment. This methodological heterogeneity, together with the complexity of tissue and circulating biomarkers, remains a major barrier to clinical validation. Moreover, few studies have simultaneously investigated both tissue-based and circulating biomarkers, comparing and correlating them within the same cohort. Most available evidence derives from studies examining either compartment in isolation, without integrating their respective clinical impact. Greater standardization and harmonization across studies will therefore be essential to facilitate biomarker comparison and support clinical translation.

Nevertheless, the integration of such high-dimensional datasets, encompassing proteomics, microbiome, metabolomics, and lipidomics, into routine clinical practice remains distant, owing to the substantial costs involved and the complexity of processing, interpreting, and standardizing large-scale multi-omics data. In the coming years, multi-omics integration (combining tissue, blood-based biomarkers, imaging, and clinical data), together with AI and machine learning methods [193,194], particularly deep learning [195,196], will play an increasingly important role in integrating heterogeneous datasets and capturing nonlinear interactions across genomes, transcriptomes, epigenomes, proteomes, metabolomes, microbiomes, and beyond. Recent reviews highlight frameworks using neural networks, graph models, and ensemble methods to generate robust feature representations that improve subtype classification, survival prediction, and therapeutic stratification beyond classical statistical approaches [191,192,193,197,198,199].

This convergence of multi-omics and machine learning is increasingly reshaping colorectal cancer research across several complementary fronts. At the level of molecular classification, integrative frameworks combining programmed cell death-related features [200] or chaperone-mediated autophagy-associated heterogeneity with dendritic cell-based immune profiling [201] have been used to derive robust prognostic subtypes and risk scores that outperform conventional single-omics classifiers. A parallel line of work has focused on predicting and explaining treatment response, particularly to radiotherapy: machine learning models trained on multi-omics data have identified candidate radio resistance biomarkers and built prognostic models in rectal cancer [202], while single-cell and multi-omics integration has similarly nominated FKBP10 as a predictor of radiotherapy outcome [203], together underscoring how these approaches can move beyond correlation toward mechanistically interpretable, functionally validated biomarkers—an effort echoed by machine learning-guided analyses identifying CPT1C as a driver of the adenoma-to-carcinoma transition [204]. Beyond established tumors, multi-omics single-cell and spatial transcriptomic approaches are also being applied earlier in the disease trajectory, mapping the immune and cellular reprogramming that accompanies the progression from inflammatory bowel disease to colorectal cancer [205]. Finally, similar computational frameworks are being translated toward diagnostic applications, as illustrated by machine learning algorithms trained on multi-omics biomarkers for the detection of microsatellite instability [206], pointing toward integration into screening and treatment-selection pipelines.

## 5. Conclusions

Tissue biomarkers remain the clinical gold standard for guiding cancer therapy but are limited by their invasiveness, sampling constraints, and inability to fully capture spatial and temporal tumor heterogeneity. These limitations have driven the development of complementary circulating biomarkers, which better reflect intra- and inter-tumor diversity and enable longitudinal monitoring of disease evolution and treatment response. However, despite their promise, the clinical implementation of circulating biomarkers remains hindered by methodological variability, pre-analytical factors, and the lack of standardized analytical thresholds.

Looking ahead, biomarker-guided cancer therapy will inevitably rely on multifactorial approaches, reflecting the biological complexity of treatment response. Immunotherapy exemplifies this challenge, as its efficacy depends on numerous interconnected factors, including tumor antigenicity, the generation of effective anti-tumor immune responses, immune cell infiltration, and the modulation of local immunosuppressive mechanisms, none of which alone is sufficient to predict clinical benefit. Accordingly, ongoing research is rapidly expanding the biomarker repertoire to include immune, metabolic, lipidomic, microbiome-derived, and other multi-omics signatures. Integrating these diverse data sources will increasingly depend on advances in computational biology and artificial intelligence to identify clinically meaningful biomarker combinations rather than relying on single markers.

Despite these exciting developments, many emerging strategies, particularly multi-omics profiling and AI-driven data integration, remain costly, analytically demanding, and dependent on specialized bioinformatic expertise. As a result, they are currently inaccessible to many healthcare systems and remain far from routine clinical implementation. Consequently, conventional, well-validated biomarkers continue to represent the most pragmatic and globally applicable standard, particularly in resource-limited settings. Realizing the full potential of precision oncology will therefore require not only harmonized protocols, prospective validation, and clinically actionable thresholds, but also the development of scalable, cost-effective, and accessible biomarker strategies that can be implemented across diverse healthcare environments, ultimately bridging the gap between biomarker discovery and equitable clinical care.

## Figures and Tables

**Table 1:** Emerging tissue biomarkers in mCRC.

Biomarker	Biological Level	Type	Value	Key Finding	Clinical Context	Validation Status	References
*SMAD4* loss	Genomic	Tumor suppressor inactivation	Prognostic (negative); Predictive (negative, ICI combinations)	Somatic SMAD4 alterations associated with shorter PFS (2.4 vs. 1.6 months) and OS (not reached vs. 5.1 months) with regorafenib + toripalimab	Refractory mCRC	Retrospective analysis of prospective trial (REGOTORI)	[30,31,32,33,34]
*POLE*/*POLD1* mutation	Genomic	DNA polymerase proofreading deficiency	Predictive (positive, ICI)	ORR 89% with anti-PD-L1 ± anti-CTLA-4 in proofreading-deficient tumors vs. 54% in dMMR/MSI-H mCRC	Refractory mCRC; independent of MMR status	Retrospective multicentre study	[35,36,37,38,39,40,41]
TMB-high (≥10–16 mut/Mb)	Genomic	High somatic mutation load; increased neoantigen burden	Predictive (positive, ICI)	ORR 38.1% in TMB ≥ 16 mut/Mb vs. 2.1% in TMB < 16 mut/Mb with atezolizumab (MyPathway)	Refractory mCRC; optimal cutoff remains debated	Prospective basket trial; CRC-specific validation ongoing	[26,38,42,43,44,45,46,47,48,49,50,51,52]
CIMP-high	Epigenomic	CpG island promoter hypermethylation; gene silencing	Prognostic (negative)	Associated with reduced OS and PFS with standard chemotherapy or anti-EGFR therapy; limited added value beyond MSI status for ICI prediction	mCRC with proximal tumors and peritoneal metastases	Retrospective cohort studies	[53,54,55,56,57,58]
Immunoediting Score (IES)	Transcriptomic	Integration of neoantigen depletion (GIE) and immune activity (ICR)	Prognostic	High IES associated with immune control; low IES associated with immune evasion and disease progression	mCRC	Retrospective; predictive value for ICI not yet validated	[59,60,61,62,63]
CMS classification	Transcriptomic	Gene expression-based molecular subtyping (CMS1-4)	Prognostic and Predictive	CMS1: benefit from pembrolizumab and bevacizumab; CMS2: benefit from anti-EGFR; CMS4: benefit from regorafenib + nivolumab (REGONIVO)	All lines of mCRC treatment	Retrospective analyses of prospective trials; limited by spatial heterogeneity	[63,64,65,66,67,68,69,70,71,72,73]
IMMETCOLS (IMC1-3)	Transcriptomic	Immunometabolic subtyping of mCRC	Predictive (investigational)	IMC1 (mesenchymal): inflamed TME, potential benefit from ICI + MCT1 inhibitors; IMC3 (epithelial): high OXPHOS, potential benefit from OXPHOS inhibitors	mCRC specifically	Single-cohort transcriptomic study; prospective validation absent	[74,75]
PD-L1/PD-1 expression	Cellular	Immune checkpoint expression on tumor and immune cells	Predictive (limited in MSS CRC)	Low ORRs with anti-PD-1/PD-L1 in MSS mCRC regardless of PD-L1 expression; high PD-1+ T cells without TH17 infiltration may identify MSI-H-like MSS tumors	MSS mCRC	Meta-analyses; no prospective validation as standalone biomarker	[76,77,78,79,80,81,82,83,84,85,86,87,88,89,90]
Immunoscore (IS)	Cellular	Density of CD3+ and CD8+ T cells in tumor centre and invasive margin	Prognostic (strong); Predictive (emerging)	High IS associated with reduced metastatic burden and improved survival; predictive benefit from ICI suggested in MSS mCRC (POCHI trial)	All stages; mCRC across lesions	Internationally validated (prognostic); prospective predictive validation ongoing	[63,91,92,93,94,95,96]
Biopsy-adapted Immunoscore (ISb)	Cellular	CD3+ and CD8+ T cell density in biopsy core; spatial ISb_20 variant	Prognostic and Predictive	ISb-high associated with improved PFS (HR = 0.24) and OS in AVETUXIRI; ISb_20 further refined stratification	Metastatic setting; pre-treatment biopsies	Prospective (AVETUXIRI); validation in larger cohorts warranted	[63,97,98]
Immunoscore-IC	Cellular	CD8+ T cell density, spatial organization, and PD-L1+ cell density	Predictive (emerging, ICI)	Greater benefit from atezolizumab in IS-IC-high MSS tumors in AtezoTRIBE; not confirmed in ANICCA-Class II	MSS mCRC; 1st-line combination immunotherapy	Phase II exploratory analyses; conflicting results across trials	[33,99,100,101]
Immunoactivation score	Cellular	Integration of lymphocyte, checkpoint, and monocyte markers (12 parameters)	Predictive (anti-EGFR maintenance)	Immunoactivation-high patients showed improved PFS (HR = 0.50) and OS (HR = 0.54) with panitumumab maintenance	RAS wild-type mCRC maintenance	Prospective (PanaMa trial); single-trial evidence	[102]
Cancer-associated fibroblasts (CAFs)/MFAP2	Cellular	Stromal remodelling; CD8+ T cell suppression via MFAP2	Prognostic (negative)	High FAP1+ CAF infiltration associated with lymph node metastasis, chemoresistance, and immunotherapy resistance; MFAP2 linked to advanced disease and poor outcome	mCRC	Retrospective; mechanistic evidence from preclinical models	[74,103,104,105,106,107,108,109,110]
Tumor microbiome (Fn, Bf, Prevotella)	Cellular/Microbial	Intratumoral bacteria modulating immune cytotoxicity and treatment response	Prognostic (negative, Fn/Bf); Predictive (investigational)	Fn associated with poor chemotherapy response, lower CD8+ T cell infiltration, and potential modulation of PD-L1; shared between primary tumors and metastases	mCRC; rectal cancer post-chemoradiotherapy	Retrospective/correlative; no prospective ICI biomarker validation	[111,112,113,114,115,116,117,118,119]

Abbreviations: TMB, tumor mutational burden; dMMR, deficient mismatch repair; MSI-H, microsatellite instability-high; MSS, microsatellite stable; ICI, immune checkpoint inhibitor; GIE, genetic immunoediting; ICR, immunologic constant of rejection; CMS, consensus molecular subtypes; IMMETCOLS, immune-metabolic colorectal signature; IMC, immune-metabolic cluster; OXPHOS, oxidative phosphorylation; TME, tumor microenvironment; MCT1, monocarboxylate transporter 1; PD-1, programmed cell death protein 1; PD-L1, programmed death-ligand 1; MDSC, myeloid-derived suppressor cell; Treg, regulatory T cell; NK, natural killer; IS, Immunoscore; ISb, biopsy-adapted Immunoscore; CAF, cancer-associated fibroblast; FAP1, fibroblast activation protein 1; MFAP2, microfibrillar-associated protein 2; Fn, *Fusobacterium nucleatum*; Bf, *Bacteroides fragilis*; ORR, objective response rate; PFS, progression-free survival; OS, overall survival; HR, hazard ratio; TCGA, The Cancer Genome Atlas.

## Data Availability

No new data were created or analyzed in this study. Data sharing is not applicable to this article.

## Abbreviations

The following abbreviations are used in this manuscript:

AUC	Area under the curve
BRAF	B-Raf proto-oncogene
CAF	Cancer-associated fibroblast
CDR3	Complementarity-determining region 3
CEA	Carcinoembryonic antigen
cfDNA	Cell-free DNA
CIMP	CpG island methylator phenotype
CISIG	Circulating inflammatory signature
CMS	Consensus molecular subtypes
CRC	Colorectal cancer
CT	Computed tomography
CTACK	Cutaneous T-cell-attracting chemokine
CTLA-4	Cytotoxic T-lymphocyte-associated protein 4
ctDNA	Circulating tumor DNA
CTC	Circulating tumor cell
CXCL10	C-X-C motif chemokine ligand 10
CCL27	C-C motif chemokine ligand 27
DC	Dendritic cell
DFS	Disease-free survival
dMMR	Deficient mismatch repair
ECM	Extracellular matrix
EGFR	Epidermal growth factor receptor
EMT	Epithelial–mesenchymal transition
FAP1	Fibroblast activation protein 1
FGF-2	Fibroblast growth factor 2
Fn	*Fusobacterium nucleatum*
Bf	*Bacteroides fragilis*
GIE	Genetic immunoediting
HER2	Human epidermal growth factor receptor 2
HR	Hazard ratio
ICI	Immune checkpoint inhibitor
ICR	Immunologic constant of rejection
IDO1	Indoleamine-2,3-dioxygenase-1
IES	Immunoediting score
IFN-γ	Interferon-gamma
IHC	Immunohistochemistry
IL	Interleukin
IMC	Immune-metabolic cluster
IMMETCOLS	Immune-metabolic colorectal signature
ISb	Biopsy-adapted Immunoscore
KRAS	Kirsten rat sarcoma viral proto-oncogene
LAG-3	Lymphocyte-activation gene 3
LDH	Lactate dehydrogenase
LMR	Lymphocyte-to-monocyte ratio
LOXL2/3	Lysyl oxidase-like protein 2/3
MCT1	Monocarboxylate transporter 1
MDSC	Myeloid-derived suppressor cell
MEK	Mitogen-activated protein kinase kinase
MET	Mesenchymal–epithelial transition factor
MFAP2	Microfibril-associated protein 2
MHC-I	Major histocompatibility complex class I
MMR	Mismatch repair
mCRC	Metastatic colorectal cancer
MSI-H	Microsatellite instability-high
MSS	Microsatellite stable
NGS	Next-generation sequencing
NK	Natural killer
NKT	Natural killer T cell
NLR	Neutrophil-to-lymphocyte ratio
NRAS	Neuroblastoma RAS viral proto-oncogene
NSCLC	Non-small cell lung cancer
ORR	Objective response rate
OS	Overall survival
OXPHOS	Oxidative phosphorylation
PBMC	Peripheral blood mononuclear cell
PD-1	Programmed cell death protein 1
PD-L1	Programmed death-ligand 1
PFS	Progression-free survival
pMMR	Proficient mismatch repair
POLE	DNA polymerase epsilon
POLD1	DNA polymerase delta 1
RAS	Rat sarcoma viral proto-oncogene
RFS	Recurrence-free survival
SCGF-β	Stem cell growth factor beta
SLD	Sum of longest diameters
SMAD4	SMAD family member 4
TCR	T cell receptor
TDO2	Tryptophan-2,3-dioxygenase
TGF-β	Transforming growth factor beta
TH1	Type 1 helper T cell
TH17	Type 17 helper T cell
TIMP1	Tissue inhibitor of metalloproteinases 1
TMB	Tumor mutational burden
TME	Tumor microenvironment
TNM	Tumor-node-metastasis
Treg	Regulatory T cell
VEGF	Vascular endothelial growth factor
WES	Whole exome sequencing
WGS	Whole genome sequencing
WT	Wild-type
